# Supplementary figures and images for: Novel inflammatory cell infiltration scoring system to investigate healthy and footrot affected ovine interdigital skin
Source: PeerJ. 2018 Jul 2;6:e5097. doi: 10.7717/peerj.5097 (PMC6033080; doi:10.7717/peerj.5097)

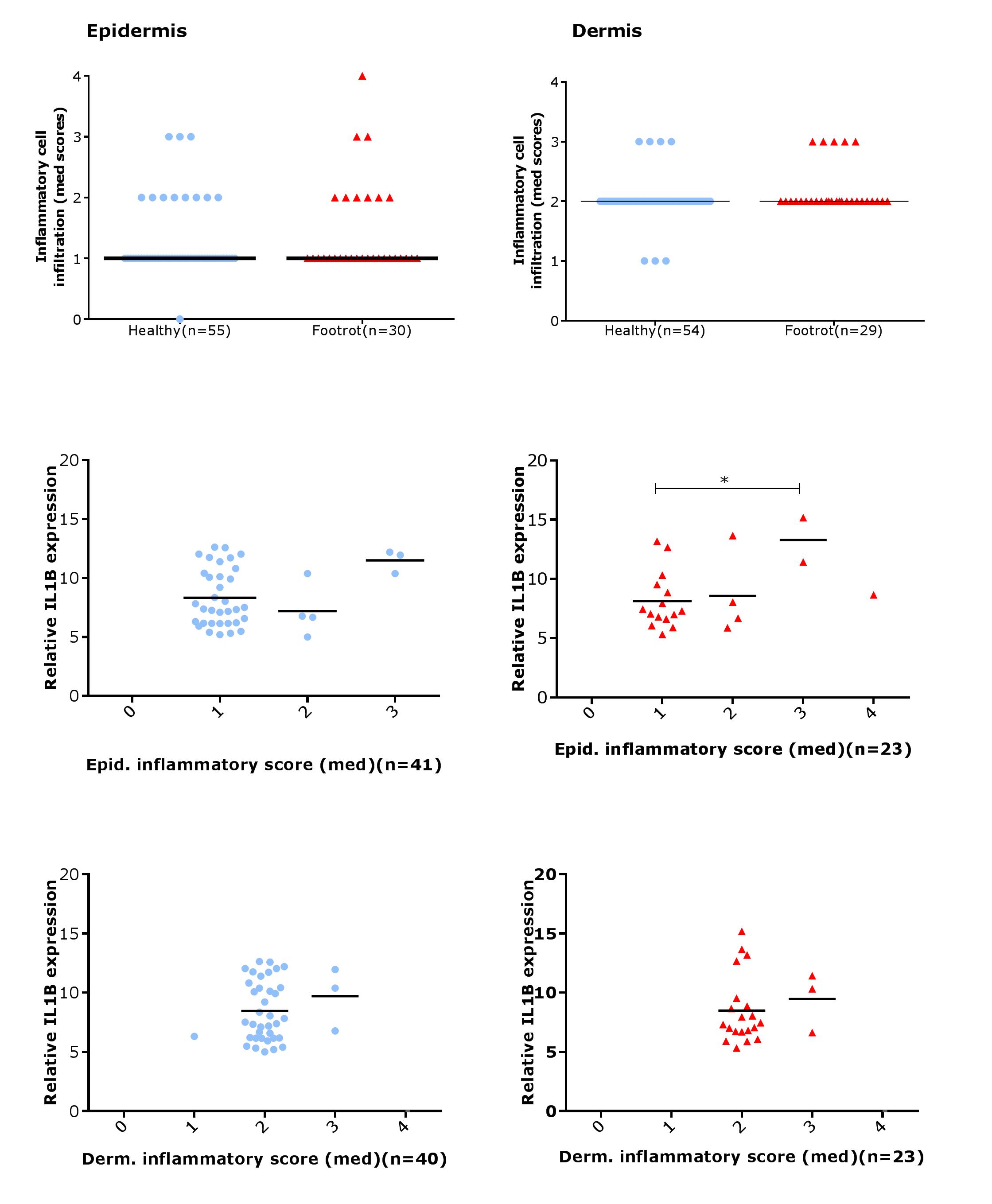

Supplement: Figure S1 — Correlation between epidermal and dermal median inflammatory scores from healthy (n = 30) and footrot samples (n = 18 epidermis and 17 dermis) in comparison to D. nodosus levels. Statistical analysis: Pearson correlation *** P < 0.0001. [file peerj-06-5097-s003.jpg]

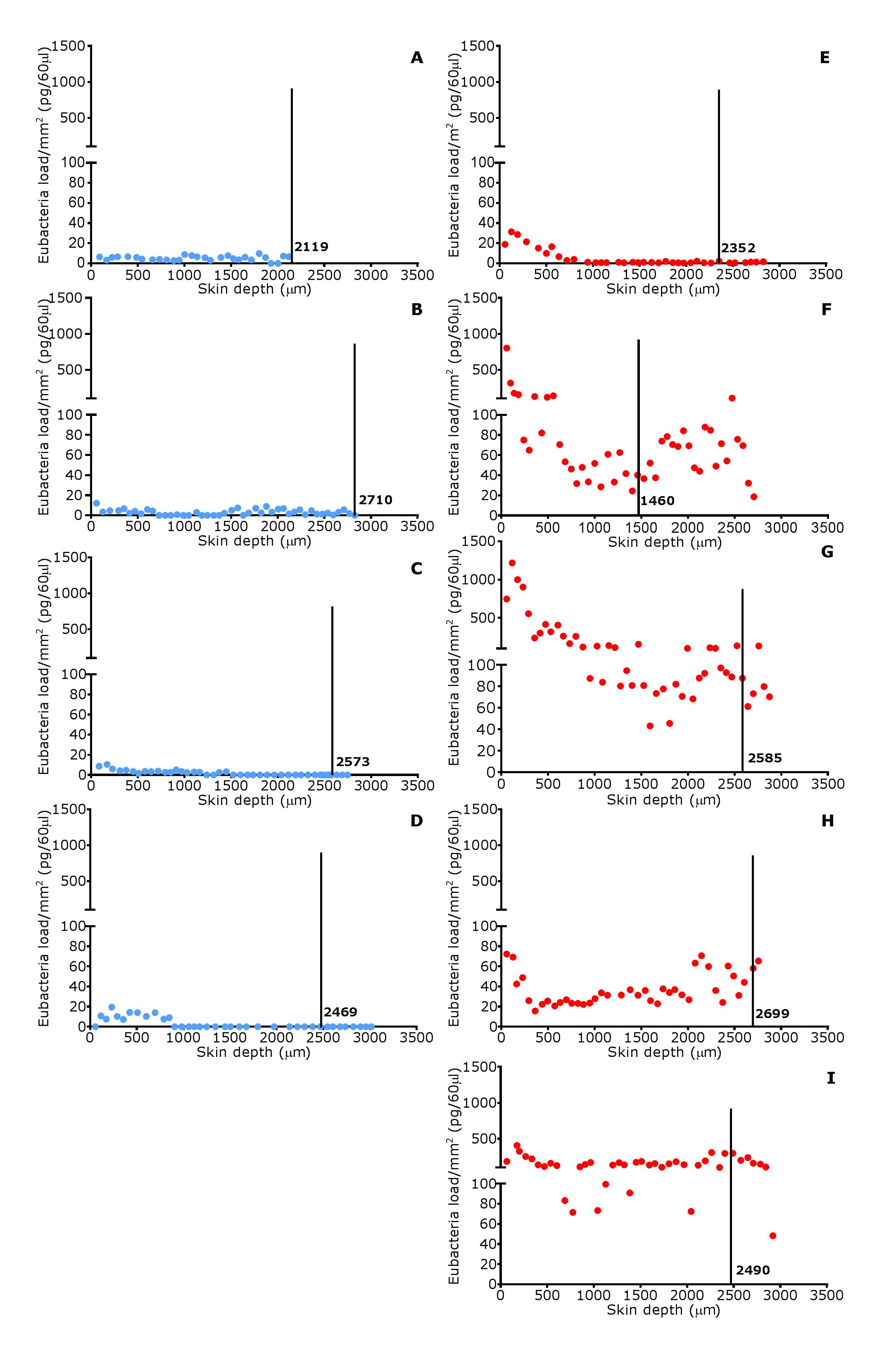

Supplement: Figure S2 — Eubacterial load presented in individual sections at progressive depths of biopsy samples in four healthy samples (A–D) and five footrot affected samples (E–I). Black vertical lines represent depth (µm) of follicles. [file peerj-06-5097-s004.jpg]
